# Modern contraceptive utilization and associated factors among reproductive-age women in Ethiopia: evidence from 2016 Ethiopia demographic and health survey

**DOI:** 10.1186/s12905-020-00923-9

**Published:** 2020-03-26

**Authors:** Mamo Nigatu Gebre, Zerihun Kura Edossa

**Affiliations:** Department of Epidemiology, Faculty of Public Health, Institute of Health, Jimma University, Jimma, Ethiopia

**Keywords:** Modern contraceptive method, Reproductive age women, 2016 EDHS, Ethiopia

## Abstract

**Background:**

Modern contraceptive methods enable couples to enjoy sexual intercourse without fear of the risk of pregnancy at any desired time. The evidence from different studies done in Ethiopia on modern contraceptive method utilization was highly varied and not conclusive. Therefore, the current study aims to study the magnitude of modern contraceptive utilization and associated factors among reproductive-age women in Ethiopia based on 2016 EDHS data.

**Method:**

A nationally representative 2016 EDHS data collected between January 18/ 2016 to June 27/2016 were used. Descriptive studies and logistic regression models were used to summarize descriptive data and measure statistical association respectively. Adjusted odds ratio and confidence interval were respectively used to measure association and its statistical significance. Finally, statistical significance was declared using a confidence interval.

**Result:**

In the current study, the overall modern contraceptive utilization among reproductive-age women in Ethiopia was 3203 (20.42%). The injectable contraceptive method was the most commonly used modern contraceptive method, 1886(58.88%) followed by implant/Norplant, 779 (24.32%). The results of multivariable logistic regression showed that age, residence, region, woman’s occupation, number of living children, husband’s education, age at first sexual intercourse, husband’s desire for more children, wealth index and watching TV were independently associated to modern contraceptive utilization among reproductive-age women in Ethiopia.

**Conclusions:**

The magnitude of modern contraceptive utilization among reproductive-age women in Ethiopia in the current study is unexpectedly low. Age, residence, region, woman’s occupation, number of living children, husband’s education, age at first sexual intercourse, husband’s desire for more children, wealth index and watching TV were independent predictors of modern contraceptive use among reproductive-age women in Ethiopia. Any intervention strategy that promotes modern contraceptive method utilization should consider these factors for its better success.

## Background

Access to safe and modern contraceptive methods contributes to more freedom, independence of women and gender equity and is, therefore, a cornerstone of women’s rights and their sexual and reproductive health [1]. Contraceptive use helps couples and individuals realize their basic right to decide freely and responsibly when and how many children to have. The growing use of contraceptive methods has resulted in not only improvements in health-related outcomes such as reduced maternal mortality and infant mortality, but also improvements in schooling and economic outcomes, especially for girls and women [2].

In developing countries, two hundred twenty-two million women who want to delay or avoid pregnancy are not using any method of family planning [3]. Worldwide, the prevalence of unmet need among married or in-union women is as high as 10%, whereas, it is doubled in the Africa region [2]. Ethiopia is still among countries with low contraceptive utilization rates even though considerable improvements have been made in the last decades [4].

Modern contraceptive methods enable couples to enjoy sex without fear of the risk of pregnancy at any desired time [5]. Modern contraceptive methods account for more than 90% of the contraceptive use worldwide. Globally in 2017, 58% of married or in-union women of reproductive age were using a modern method which accounts for 92% of all contraceptive users [2].

Utilizing contraceptive methods prevent sexually transmitted diseases in addition to avoiding unwanted pregnancies, unintended birth, abortion, miscarriages and maternal death [3].

A community-based cross-sectional study done in Ho district of west Ghana showed that 89.8% of married women used modern contraceptives, and the injectable contraceptive method was the most used [6]. To the contradict, another study was done in the same country (Ghana) showed a prevalence of only 21% [7]. A community-based cross-sectional study done on reproductive-age women in Yaoundé-Cameroon showed that 58.9% used a modern contraceptive method [8]. Different studies done in Ethiopia [9–14] showed extremely variable results in the prevalence of modern contraceptive use which ranged from 20.8% from the study done at Bale Zone, South East Ethiopia [9] to 71.9% from the community-based cross-sectional study done at western Ethiopia [14]. Studies done in different parts of Ethiopia also showed that the injectable contraceptive method was the most used modern contraceptive method [9, 11, 12, 14].

Religious opposition, desire for more children, fear of side effects, husband opposition, inter-spousal discussion, Perceived husband approval, discussion with HEW, perceived cultural acceptability, Women’s educational status, gravidity, postnatal care utilization, age, Husband’s education, marital status, monthly income, fertility, media exposure, number of living children, woman’s decision making autonomy, family planning counseling and Having postnatal care were some of the factors independently associated with modern contraceptive utilization [6, 8–15].

Evidence from the aforementioned studies done in Ethiopia was highly varied and the results are inconclusive. Therefore, the current study aims to study the magnitude of modern contraceptive utilization and its predictors among reproductive-age women in Ethiopia based on the 2016 EDHS data. The current study will provide up-to-date evidence for policymakers and other stakeholders working on family planning in Ethiopia and similar settings to solve problems related to modern contraceptive utilization based on evidence.

## Methods

### Population

All reproductive age women in Ethiopia, based on 2016 EDHS were included in the study.

### Data source

The current study used data extracted from 2016 EDHS which was stratified into urban and rural areas and yielded twenty-one sampling strata. A two-stage sampling technique was done to select representative samples of independent enumeration areas in each stratum. In the first stage, 202 urban areas and 443 rural areas were selected using probability proportional to the size of enumeration area. Lists of households were prepared from the selected enumeration areas and served as a sampling frame for the selection of representative households in the second stage. Lastly, a total of 18,008 households were selected for the study out of which only 17,067 households were occupied. Complete interview was obtained from 16,650 households making the response rate 98%. In those interviewed households, 16,583 women were identified and 15,683 women completed the interview making a response rate of 95%. Therefore, data for the current study came from an individual record of 15,683 reproductive-age women. For all eligible women aged 15–49 years, the woman’s questionnaire comprising five different parts including family planning was used to collect information [16].

### Statistical analysis

Statistical package for social science (SPSS) was used for statistical analysis. Descriptive studies like measures of central tendency and measures of dispersion for continuous data, and frequency count and proportion for categorical data were used to summarize descriptive data. Bivariate logistic regression was used to select candidate variables for multivariable logistic regression. In the bivariate logistic regression, a *p*-value of less than 0.2 was used as a cut of point. Variable inflation factor (VIF) was used to check multi-collinearities between candidate variables before fitting the final model. Multivariable logistic regression was used to identify independent predictors of modern contraceptive method utilization among reproductive-age women in Ethiopia and to control confounders. Adjusted odds ratio and confidence interval (CI) were respectively used to measure the association between modern contraceptive utilization and predictor variables and their statistical significance in the final model. The confidence interval was used to declare statistical significance in the final model. Hosmer and Lameshow test was used to check model fitness.

### Operational definitions

Modern contraceptive utilization: in the current study a woman was considered as modern contraceptive method utilizer if she had been using at least one of the modern contraceptives (female sterilization, male sterilization, IUCD, injectable, implants, pills, male condom, female condom, emergency contraception, and standard days method) during EDHS data collection period.

Modern contraceptive non-utilization: a woman was considered to be non-utilizer of the modern contraceptive method if she had been using traditional methods like rhythm method, lactation amenorrhea method, and withdrawal or if she had not been using any type of contraception during EDHS data collection period.

## Results

### Socio-demographic and socio-economic characteristics

**Fig. 1:**
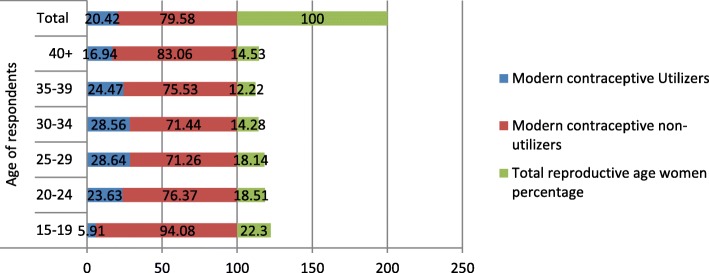
Total reproductive age women and Modern contraceptive utilization by age, 2016

**Fig. 2:**
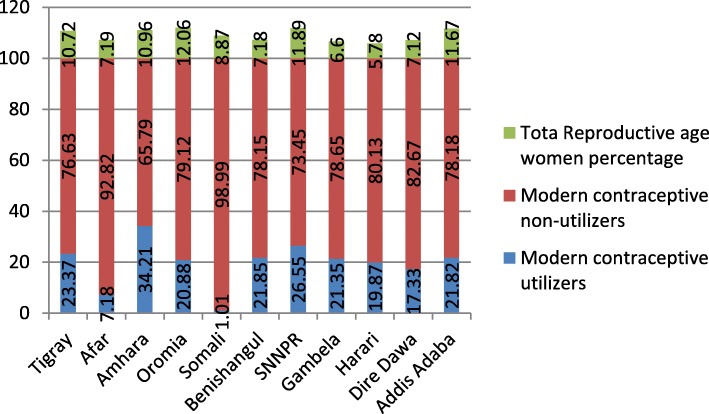
Total reproductive age women and Modern contraceptive utilization by regions of Ethiopia, 2016

**Fig. 3:**
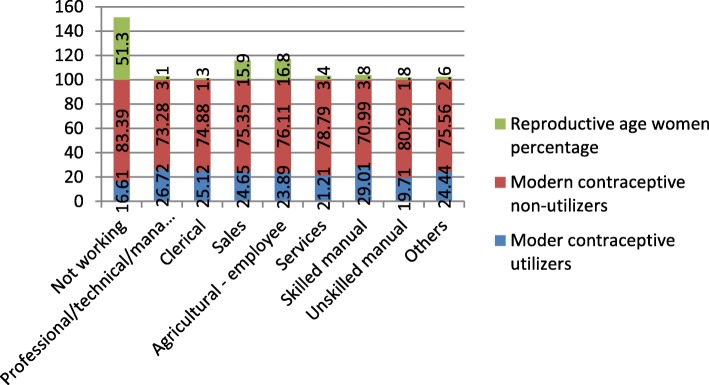
Reproductive age women percentage and modern contraceptive method utilization by occupation, 2016

In the interviewed households, 16,583 eligible women were identified for individual interviews. Interviews were completed with 15,683 women, yielding a response rate of 95%. The mean age of respondents was 27.94 (±9.16) years with the age range of 34 years. From the total 15,683 respondents interviewed, 3498 (22.3%) were within the age group of 15–19 years. Out of the total reproductive age women interviewed, 18.51% (2903) and 18.14% (2845) were found in the age groups of 25–29 and 30–34 years respectively (Fig. 1). From the total respondents, 1892 (12.06%) and 907 (5.78%) were from Oromiya and Harari region respectively (Fig. 2). Regarding the occupation of the participants, 8045 (51.3%) were not working and 211(1.3%) were clericals (Fig. 3). Regarding the residence of respondents, 10,335 (65.9%) women were rural residents. Concerning educational status, 7033(44.8%) were not educated and 4524 (46.1%) women had uneducated husbands. 7591 (63.5%) and 4094 (39.9%) reproductive age women committed first sexual intercourse and bore the first child at age of less than 18 years respectively. Regarding the economic status of the women, 5699(36.3%) women were from the richest family. Concerning decision making on contraceptive use, 2104 (70.6%) reproductive age women jointly decided with their husbands (Table 1).

**Table 1:** Socio-demographic and socio-economic characteristics of reproductive age women in Ethiopia, 2016

Socio-demographic and socio-economic characteristics	Variable Categories	Frequency (%)	Modern contraceptive utilization	
			Yes (%)	No (%)
Residence	Urban	5348 (34.1)	1257 (23.5)	4091 (76.5)
	rural	10,335 (65.9)	1946 (18.8)	8389 (81.2)
current marital status	never married	4278 (27.3)	119 (2.78)	4159 (97.2)
	married/living together	9824 (62.6)	2886 (29.3)	6938 (70.7)
	married but not together	1581 (10.1)	198 (12.5)	1383 (87.5)
Respondents educational level	No education	7033 (44.8)	1325 (18.83)	5708 (81.16)
	Primary	5213 (33.2)	1149 (22.04)	4064 (77.96)
	Secondary	2238 (14.3)	428 (19.12)	1810 (80.88)
	Higher	1199 (7.6)	301 (25.10)	898 (74.90)
Husband’s education	not educated	4524 (46.1)	905 (20.0)	3619 (80.0)
	primary	3054 (31.1)	1121 (36.7)	1933 (63.3)
	secondary	1226 (12.5)	470 (38.3)	756 (61.7)
	higher	1020 (10.4)	390 (38.3)	630 (61.7)
Wealth index	Poorest	3894 (24.8)	327 (8.4)	3567 (91.6)
	Poorer	2046 (13.1)	432 (21.1)	1614 (78.9)
	Middle	2002 (12.8)	494 (24.7)	1508 (75.3)
	Richer	2042 (13.0)	538 (26.3)	1504 (73.7)
	Richest	5699 (36.3)	1412 (24.8)	4287 (75.2)

### Prevalence of modern contraceptive use

**Fig. 4:**
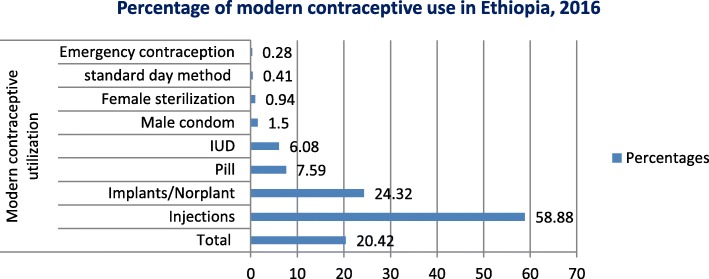
Prevalence of Modern Contraceptive Utilization in Ethiopia, 2016

The overall prevalence of modern contraceptive utilization was 3203 (20.42%). The injectable contraceptive method was the most, 1886(58.88%) commonly used modern contraceptive method followed by implant/Norplant, 779 (24.32%) (Fig. 4). From the reproductive age women aged 15–19 years only 5.91% (207) utilized modern contraceptive method whereas 28.64 and 28.56% of reproductive age women aged between 25 and 29 years and 30–34 years utilized modern contraceptive methods respectively. There was also a disparity of modern contraceptive utilization between rural and urban resident reproductive-age women; only 1946(18.8%) rural residents utilized modern contraceptive whereas 1257 (23.5%) urban residents utilized modern contraceptive. Modern contraceptive utilization was also highly varied based on marital status; only 119 (2.78%) unmarried reproductive age women utilized modern contraceptive methods whereas 2886 (29.3%) reproductive-age women who were married and living together utilized modern contraceptive method. There was also a great disparity in modern contraceptive utilization based on respondents’ wealth index; only 198(12.5%) reproductive women from poorest households utilized modern contraceptive methods whereas 538 (26.3%) women from richer households utilized modern contraceptive method. Modern contraceptive utilization was also highly varied by regions of Ethiopia; 34.21 and 1.01% reproductive age women utilized modern contraceptive methods from Amhara and Harari regions respectively.

### Individual and behavioral characteristics of reproductive age women in Ethiopia

Regarding age at first sexual intercourse and age at first childbirth, 7591 (63.5%) and 5286(51.5%) had committed their first sexual intercourse and had born their first child at age of less than 18 years and 18–24 year respectively. 5409 (34.5%) and 5539(38.4%) reproductive age women did not bear a child and did not have a living child respectively. 13,106(83.6%), 10,338(65.9%) and 10,084(64.3%) did not read the newspaper, listen to the radio and watch television. Regarding decision making on contraceptive use, 2104(70.6%) women made the decision jointly with their husbands (Table 2).

**Table 2:** Individual and behavioral related characteristics of reproductive age women in Ethiopia, 2016

Variable	Categories	Frequency (%)	Modern contraceptive utilization	
			Yes	No
Age at first sex	less 18 years	7591 (63.5)	1989 (26.2)	5602 (73.8)
	18–24	3961 (33.1)	1125 (28.4)	2836 (71.6)
	25 and above	410 (3.4)	77 (18.8)	333 (81.2)
Age at first childbirth	less 18 years	4094 (39.9)	1044 (25.5)	3050 (74.5)
	18–24	5286 (51.5)	1549 (29.3)	3737 (70.7)
	25 and above	886 (8.6)	235 (26.5)	651 (73.5)
Total children ever born	No child	5409 (34.5)	374 (6.91)	5035 (93.09)
	1–2	3678 (23.5)	1266 (34.42)	2412 (65.58)
	3–4	2683 (17.1)	785 (29.26)	1898 (70.74)
	5 and above	3913 (25.0)	778 (19.88)	3135 (80.12)
Number of living children	No child	5539 (38.4)	390 (7.04)	5149 (92.96)
	1–2	3983 (27.6)	1355 (34.02)	2628 (65.98)
	3–4	2930 (20.2)	807 (27.54)	2123 (72.46)
	5 and above	1990 (13.8)	438 (22.01)	1552 (77.10)
Frequency of reading newspaper or magazine	Not at all	13,106 (83.6)	2632 (20.08)	10,474 (79.92)
	Less than once a week	1881 (12.0)	428 (22.75)	1453 (77.25)
	At least once a week	696 (4.4)	143 (20.55)	553 (79.45)
Frequency of listening to the radio	Not at all	10,338 (65.9)	1908 (18.46)	8430 (81.54)
	Less than once a week	2644 (16.9)	623 (23.56)	2021 (76.44)
	At least once a week	2701 (17.1)	672 (24.88)	2029 (78.12)
Frequency of watching TV	Not at all	10,084 (64.3)	1820 (18.05)	8264 (81.95)
	Less than once a week	1780 (11.3)	444 (24.94)	1336 (75.06)
	At least once a week	3819 (24.4)	939 (24.59)	2880 (75.41)
Decision make on contraceptive use	Mainly respondent	726 (24.4)	705 (97.1)	21 (2.9)
	Mainly husband, partner	150 (5.0)	147 (98.0)	3 (2.0)
	Joint decision	2104 (70.6)	2034 (96.7)	70 (3.3)

### Results of bivariate analysis

Bivariate logistic regression was fitted to identify candidate variables for multivariable logistic regression. Accordingly: Age of respondent, residence, region of residence, current marital status, educational level, occupation, total children ever born, number of living children, husband’s educational level, age at first sex, husband’s desire for child, wealth index, frequency of listening to radio and frequency of watching television were candidate for multivariable logistic regression (Table 3).

**Table 3:** Bivariate analysis of the association between modern contraceptive utilization and associated factors

Characteristics	Category	Frequency (%)	Modern contraceptive utilization		COR (95%CI)	P
			Yes	No		
Age of respondent	15–19	3498 (22.3)	207	3291	.308 (.258,.369)	.000*
	20–24	2903 (18.5)	686	2217	1.517 (1.321,1.744)	.000*
	25–29	2845 (18.1)	815	2030	1.969 (1.718,2.256)	.000*
	30–34	2241 (14.3)	640	1601	1.960 (1.700,2.261)	.000*
	35–39	1917 (12.2)	469	1448	1.588 (1.366,1.847)	.000*
	40^+^	2279 (14.5)	386	1893	1	
Residence	Urban	5348 (34.1)	1257	4091	1.325 (1.223,1.435)	.000*
	rural	10,335 (65.9)	1946	8389	1	
Region	Tigray	1682 (10.7)	393	1289	1.092 (.932,1.280)	.274
	Afar	1128 (7.2)	81	1047	.277 (.215,.357)	.000*
	Amhara	1719 (11.0)	588	1131	1.863 (1.604,2.163)	.000*
	Oromia	1892 (12.1)	395	1497	.945 (.808,1.106)	.483
	Somali	1391 (8.9)	14	1377	.036 (.021,.062)	.000*
	Benishangul	1126 (7.2)	246	880	1.002 (.837,1.199)	.986
	SNNPR	1849 (11.8)	491	1358	1.295 (1.113,1.508)	.001*
	Gambela	1035 (6.6)	221	814	.973 (.808,1.171)	.771
	Harari	906 (5.8)	180	726	.888 (.729,1.082)	.240
	Dire Dawa	1131 (7.2)	196	935	.751 (.621,.908)	.003*
	Addis Adaba	1824 (11.6)	398	1426	1	
current marital status	never married	4278 (27.3)	119	4159	1	
	married/living together	9824 (62.6)	2886	6938	.200 (.158,.253)	.000*
	married but not together	1581 (10.1)	198	1383	2.905 (2.488,3.393)	.000*
Respondent educational level	No education	7033 (44.8)	1325	5708	1	
	Primary	5213 (33.2)	1149	4064	.693 (.600,.799)	.000*
	Secondary	2238 (14.3)	428	1810	.843 (.729,.976)	.022*
	Higher	1199 (7.6)	301	898	.705 (.597,.834)	.000*
Respondent’s Occupation	Not working	8045 (51.3)	1336	6709	1	
	Professional/technical/managerial	494 (3.1)	132	362	.616 (.487,.779)	.000*
	Clerical	211 (1.3)	53	158	1.127 (.833,1.526)	.437
	Sales	2495 (15.9)	615	1880	1.037 (.705,1.525)	.853
	Agricultural - employee	2637 (16.8)	630	2007	1.011 (.791,1.293)	.928
	Services	528 (3.4)	112	416	.971 (.760,1.240)	.811
	Skilled manual	593 (3.8)	172	421	.832 (.611,1.134)	.244
	Unskilled manual	279 (1.8)	55	224	1.263 (.946,1.686)	.113*
	Others	401 (2.6)	98	303	.759 (.523,1.102)	.147*
Total children ever born	No child	5409 (34.5)	374	5035	.299 (.263,.341)	
	1–2	3678 (23.5)	1266	2412	2.115 (1.906,2.347)	.000*
	3–4	2683 (17.1)	785	1898	1.667 (1.486,1.869)	.000*
	5 and above	3913 (25.0)	778	3135	1	.000*
Number of living children	No child	5539 (38.4)	390	5149	.268 (.232,.311)	
	1–2	3983 (27.6)	1355	2628	1.827 (1.613,2.070)	.000*
	3–4	2930 (20.2)	807	2123	1.347 (1.179,1.539)	.000*
	5 and above	1990 (13.8)	438	1552	1	.000*
Husband’s education	not educated	4524 (46.1)	905	3619	1	
	primary	3054 (31.1)	1121	1933	.404 (.349,.467)	.000*
	secondary	1226 (12.5)	470	756	.937 (.809,1.084)	.381
	higher	1020 (10.4)	390	630	1.004 (.847,1.191)	.961
Age at first sex	less 18 years	7591 (63.5)	1989	5602	1.535 (1.192,1.978)	.000*
	18–24	3961 (33.1)	1125	2836	1.716 (1.326,2.219)	.000*
	25 and above	410 (3.4)	77	333	1	
Age at first childbirth	less 18 years	4094 (39.9)	1044	3050	.948 (.804,1.118)	.527
	18–24	5286 (51.5)	1549	3737	1.148 (.978,1.348)	.091*
	25 and above	886 (8.6)	235	651	1	
Husband’s desire for children	Both want the same	3894 (39.8)	1364	2530	1	.000*
	Husband wants more	2641 (27.0)	610	2031	1.612 (1.444,1.800)	.456
	Husband wants fewer	634 (6.5)	225	409	.898 (.792,1.019)	.000*
	Don’t know	2626 (26.8)	658	1968	1.645 (1.367,1.980)	
Wealth index	Poorest	3894 (24.8)	327	3567	1	
	Poorer	2046 (13.1)	432	1614	.278 (.245,.316)	.000*
	Middle	2002 (12.8)	494	1508	.813 (.719,.918)	.001*
	Richer	2042 (13.0)	538	1504	.995 (.884,)	.928
	Richest	5699 (36.3)	1412	4287	1.086 (.968,1.219)	.161*
The decision makes on contraceptive use	Mainly respondent	726 (24.4)	705	21	1.155 (.704,1.896)	.568
	Mainly husband, partner	150 (5.0)	147	3	1.686 (.525,5.421)	.380
	Joint decision	2104 (70.6)	2034	70	1	
Frequency of reading newspaper or magazine	Not at all	13,106 (83.6)	2632	10,474	1	
	Less than once a week	1881 (12.0)	428	1453	.972 (.805,1.174)	.766
	At least once a week	696 (4.4)	143	553	1.139 (.920,1.410)	.231
Frequency of listening to the radio	Not at all	10,338 (65.9)	1908	8430	1	
	Less than once a week	2644 (16.9)	623	2021	.683 (.618,.756)	.000*
	At least once a week	2701 (17.1)	672	2029	.931 (,.821,1.055)	.261
Frequency of watching TV	Not at all	10,084 (64.3)	1820	8264	1	
	Less than once a week	1780 (11.3)	444	1336	.675 (.618,.739)	.000*
	At least once a week	3819 (24.4)	939	2880	1.019 (.895,1.161)	.72

^1^ Reference

*Statistically significant

### Predictors of modern contraceptive use among reproductive-age women in Ethiopia, 2016

A multivariable logistic regression model was fitted to identify independent predictors of modern contraceptive method utilization among reproductive-age women in Ethiopia. Accordingly; age, place of residence, region of residence, occupation, number of living children, Husband’s education, age at first sex, husband’s desire for children, wealth index and frequency of watching television were independently associated with modern contraceptive method utilization.

Woman whose age was between 15 and 19, 20–24, 25–29, 30–34 and 35–39 was nearly 1.9 [AOR = 1.911(1.301,2.806)], 2.4 [AOR = 2.389(1.853,3.081)], 2.2 [2.196(1.782,2.706)], 1.9[AOR = 1.938(1.589,2.363)] and 1.8 [1.797(1.464,2.207)] times more likely to utilize modern contraceptive method as compared to woman whose age was 40 year and above.

The odds of modern contraceptive utilization among urban resident women was nearly 1.5 [AOR = 1.5129 (1.204, 1.900)] times more likely than the odds of modern contraceptive use among rural resident woman.

A Women who resides in Tigray, Amhara and SNNPR regions of Ethiopia was nearly 1.4 [AOR = 1.370 (1.041, 1.804)], 3 [AOR = 2.890 (2.181, 3.831)] and 2 [AOR = 1.95 (1.484, 2.565)] times more likely to utilize modern contraceptive method as compared to woman who resides in Addis Ababa respectively. A woman who resides in Afar and Somali region of Ethiopia was nearly 55% [(AOR = .446 (.310, .643)] and 93% [(AOR = .070, (.038, .130)] times less likely to utilize modern contraceptive method as compared to a woman who resides in Addis Ababa respectively.

A woman who earns her life by selling goods, agricultural employment, skilled manual working, and other different jobs was nearly 1.3 [AOR = 1.336(1.136,1.572)], 1.2[1.192(1.022,1.390)], 1.4[AOR = 1.425(1.085,1.872)] and 1.6 [AOR = 1.331(1.039,1.706)] times more likely to utilize modern contraceptive method as compared to a woman who did not have any job respectively.

A woman who had no child or who had 1–2 child/children was nearly 75% [AOR = .255(.117,.556)] and 34% [AOR = .658(.455,.952)] times less likely to utilize modern contraceptive methods as compared to a woman who had born 5 or more children.

A woman whose husband had completed primary education was nearly 1.4 [AOR = 1.347(1.173, 1.546)] times more likely to utilize modern contraceptive methods as compared to a woman whose husband was not educated.

A woman who had started her first sexual intercourse at an age of less than 18 years was nearly 1.6 [AOR = 1.582(1.054,2.373)]times more likely to utilize modern contraceptive methods as compared to a woman who started her first sexual intercourse at an age of 25 or more year respectively.

The odds of modern contraceptive utilization among women whose husbands want more children was nearly 25% [AOR = .749(.653,.860)]times less likely than the odds of modern contraceptive utilization among women who want the same number of children with their husbands. The odds of modern contraceptive utilization among women where neither husbands’ nor women’s desire for more children is not known was nearly 32% [AOR = .677(.590, .776)] times less likely than the odds of modern contraceptive utilization among women where women and husbands want the same number of children.

A woman who was from the richest, a richer, middle and poorer household was nearly 3.5 [AOR = 3.462(2. 672, 4.485], 3 [AOR = 3.034(2.471, 3.726)], 2.5 [AOR = 2.471(2.021, 3.022)] and 2 [AOR = 1.929(1.580, 2.356)] times more likely to utilize modern contraceptive method as compared to reproductive age woman from poorest household respectively.

A woman who watches television at least once a week and less than once a week was nearly 1.3[AOR = 1.259(1.036,1.531)] and 1.4[AOR = 1.368(1.110,1.687)] times more likely to utilize modern contraceptive method as compared to a woman who does not watch a television respectively (Table 4).

**Table 4:** Independent predictors of modern contraceptive utilization among reproductive-age women in Ethiopia, 2016

Characteristics	Category	Frequency (%)	COR (95%CI)	AOR (95% CI)
Age of respondent	15–19	3498 (22.3)	.308 (.258,.369)	1.911 (1.301,2.806)*
	20–24	2903 (18.5)	1.517 (1.321,1.744)	2.389 (1.853,3.081) *
	25–29	2845 (18.1)	1.969 (1.718,2.256)	2.196 (1.782,2.706) *
	30–34	2241 (14.3)	1.960 (1.700,2.261)	1.938 (1.589,2.363) *
	35–39	1917 (12.2)	1.588 (1.366,1.847)	1.797 (1.464,2.207) *
	40^+^	2279 (14.5)	1	1
Residence	Urban	5348 (34.1)	1.325 (1.223,1.435)	1.512 (1.204,1.900) *
	rural	10,335 (65.9)	1	1
Region	Tigray	1682 (10.7)	1.092 (.932,1.280)	1.370 (1.041,1.804) *
	Afar	1128 (7.2)	.277 (.215,.357)	.446 (.310,.643) *
	Amhara	1719 (11.0)	1.863 (1.604,2.163)	2.890 (2.181,3.831) *
	Oromia	1892 (12.1)	.945 (.808,1.106)	1.294 (.986,1.699)
	Somali	1391 (8.9)	.036 (.021,.062) *	.070 (.038,.130) *
	Benishangul	1126 (7.2)	1.002 (.837,1.199)	1.252 (.925,1.694)
	SNNPR	1849 (11.8)	1.295 (1.113,1.508)	1.951 (1.484,2.565) *
	Gambela	1035 (6.6)	.973 (.808,1.171)	1.103 (.824,1.478)
	Harari	906 (5.8)	.888 (.729,1.082)	.777 (.584,1.034)
	Dire Dawa	1131 (7.2)	.751 (.621,.908)	.761 (.573,1.011)
	Addis Adaba	1824 (11.6)	1	1
Respondent’s Occupation	Not working	8045 (51.3)	1	1
	Professional/technical/managerial	494 (3.1)	.616 (.487,.779)	1.095 (.745,1.608)
	Clerical	211 (1.3)	1.127 (.833,1.526)	1.287 (.739,2.240)
	Sales	2495 (15.9)	1.037 (.705,1.525)	1.336 (1.136,1.572) *
	Agricultural - employee	2637 (16.8)	1.011 (.791,1.293)	1.192 (1.022,1.390) *
	Services	528 (3.4)	.971 (.760,1.240)	1.229 (.868,1.740)
	Skilled manual	593 (3.8)	.832 (.611,1.134)	1.425 (1.085,1.872) *
	Unskilled manual	279 (1.8)	1.263 (.946,1.686)	1.382 (.858,2.227)
	Others	401 (2.6)	.759 (.523,1.102)	1.570 (1.085,2.271) *
Total children ever born	No child	5409 (34.5)	.299 (.263,.341)	1.711 (1.168,2.505) *
	1–2	3678 (23.5)	2.115 (1.906,2.347)	1.331 (1.039,1.706) *
	3–4	2683 (17.1)	1.667 (1.486,1.869)	1.421 (1.123, 2.341) *
	5 and above	3913 (25.0)	1	1
Number of living children	No child	5539 (38.4)	.268 (.232,.311)	.255 (.117,.556) *
	1–2	3983 (27.6)	1.827 (1.613,2.070)	.658 (.455,.952) *
	3–4	2930 (20.2)	1.347 (1.179,1.539)	.792 (.616,1.018)
	5 and above	1990 (13.8)	1	1
Husband’s education	not educated	4524 (46.1)	1	1
	primary	3054 (31.1)	.404 (.349,.467)	1.347 (1.173,1.546) *
	secondary	1226 (12.5)	.937 (.809,1.084)	1.069 (.872,1.309)
	higher	1020 (10.4)	1.004 (.847,1.191)	.956 (.749,1.221)
Age at firs sex	less 18 year	7591 (63.5)	1.535 (1.192,1.978)	1.582 (1.054,2.373) *
	18–24 year	3961 (33.1)	1.716 (1.326,2.219)	1.428 (.964,2116)
	25 and above	410 (3.4)	1	1
Husband’s desire for children	Both want the same	3894 (39.8)	1	1
	Husband wants more	2641 (27.0)	1.612 (1.444,1.800)	.749 (.653,.860) *
	Husband wants fewer	634 (6.5)	.898 (.792,1.019)	.949 (.770,1.170)
	Don’t know	2626 (26.8)	1.645 (1.367,1.980)	.677 (.590,.776) *
Wealth index	Poorest	3894 (24.8)	1	1
	Poorer	2046 (13.1)	.278 (.245,.316)	1.929 (1.580,2.356) *
	Middle	2002 (12.8)	.813 (.719,.918)	2.471 (2.021,3.022) *
	Richer	2042 (13.0)	.995 (.884,)	3.034 (2.471,3.726) *
	Richest	5699 (36.3)	1.086 (.968,1.219)	3.462 (2672,4.485) *
Frequency of watching TV	Not at all	10,084 (64.3)	1	1
	Less than once a week	1780 (11.3)	.675 (.618,.739)	1.259 (1.036,1.531) *
	At least once a week	3819 (24.4)	1.019 (.895,1.161)	1.368 (1.110,1.687) *

^1^ Reference

*Statistically significant

## Discussions

The overall prevalence of modern contraceptive utilization among reproductive-age women in Ethiopia was 3203 (20.42%). The finding is almost consistent with the result of the 2014 Ghana Demographic and Health Surveys secondary data analysis where the prevalence of modern contraceptive utilization was 21.53% [17]. It was also consistent with cross-sectional studies done in Ghana and Ethiopia in 2016 where 21 and 20.8% of reproductive age women used a modern contraceptive method respectively [9, 18]. But the current prevalence is higher than the results of secondary data analysis of Nigeria (10.3%) and Mali (15.3%) Demographic and Health Survey which were conducted in 2013 and 2012 respectively [19, 20], and lower than the results of secondary data analysis of Burkina Faso (24%) and Afghanistan (25.5%) Demographic and Health Survey which were conducted in 2012 and 2015respectively [20, 21]. A cross-sectional multi-country analysis of Demographic and Health Surveys (DHS) conducted between 2008 and 2016 in 52 low and middle-income countries (LMICs) and taking 6857 representative sample from Ethiopia also showed that a prevalence of modern contraceptive method utilization among Ethiopian young reproductive-age women aged between 15 and 24 years was 12.1% which is by far lower than the result from the current study [15]. The current prevalence is also lower than the results of other studies done in Ethiopia at a different time [10–14]. The discrepancy might have occurred due to a difference in awareness of modern contraceptive methods. The difference might also be due to the socio-cultural difference between the different countries which might have a paramount effect on contraceptive utilization. The current study revealed that the injectable contraceptive method was the most commonly used contraceptive method which was consistent with the results of different studies done in Ethiopia [9, 11, 12, 14].

In the current study, age was an independent predictor of modern contraceptive method utilization among reproductive-age women in Ethiopia. A woman whose age was between 15 and 19, 20–24, 25–29, 30–34 and 35–39 was nearly 1.9, 2.4, 2.2, 1.9 and 1.8 times more likely to utilize modern contraceptive methods as compared to a woman whose age was 40 year and above. Results from secondary data analysis of three consecutive Bangladesh Demographic and Health Surveys also showed that a woman whose age was from 40 to 49 years less likely practiced modern contraceptive utilization which is consistent with the current study [22]. The current result was also consistent with the results of two cross-sectional studies done in Northwest and Western Ethiopia where reproductive age women aged 35–49 years and > 44 years respectively were less likely utilized modern contraceptive method [10, 14]. Another cross-sectional study done in Cameroon in 2014 and 2015 also showed that age > 30 years was negatively associated with modern contraceptive method utilization which was also consistent with the current study [8]. This might be due to the reason that reproductive-age women from low-income countries become more economically stable at their late reproductive age and might not want to utilize contraceptive methods to bear more children. But the current result is in contrast with the results of secondary data analysis of Nigeria (2013) and Afghanistan (2012) Demographic and Health survey where women greater than 40 years age more likely utilized modern contraceptive method [19, 23]. The difference might have occurred due to the difference in the socio-economic and socio-cultural differences between the countries.

Residence was also independently associated with modern contraceptive utilization in the current study. The odds of modern contraceptive utilization among urban resident reproductive-age woman was nearly 1.5 times more likely than the odds of modern contraceptive utilization among rural resident woman. The finding is consistent with the results of secondary data analysis of Indian, Afghanistan, Nigeria and Bangladesh Demographic and Health surveys where urban resident women more likely utilized modern contraceptive methods than rural resident women [19, 21, 22, 24]. This could be due to different reasons. Urban women are more educated, have better income, have better access to the health facility, and better media access than rural women which have a positive impact on modern contraceptive utilization. Rural women also need more children to help them with fieldwork which has a negative effect on their modern contraceptive method utilization [14, 19, 21, 23, 25, 26]. But the result of secondary data analysis of the 2003–2014 Ghana Demographic and Health Surveys showed that rural resident women more likely utilized modern contraceptive methods than urban resident women which are in contrary to the results of the current study [17]. The difference could be due to the difference in awareness, availability, and accessibility of modern contraceptives for rural women.

The study also showed that the region of residence was an independent predictor of modern contraceptive method utilization among reproductive-age women. A woman who resides in Tigray, Amhara and SNNPR regions of Ethiopia was nearly 1.4,3 and 2 times more likely utilized modern contraceptive method as compared to a woman who resides in Addis Ababa respectively. A woman who resides in the Afar and Somali region of Ethiopia was nearly 55 and 93% times less likely to utilize modern contraceptive methods as compared to a woman residing in Addis Ababa. The finding is consistent with the results of secondary data analysis of the 2013 Nigerian Demographic and Health Survey (NDHS) [19, 25] analyzed by independent researchers.

In the current study, the occupation was independently associated with modern contraceptive utilization. Reproductive age women who earn their lives by selling goods, agricultural employment, skilled manual working, and other different jobs were nearly 1.3, 1.2, 1.4 and 1.6 times more likely to utilize modern contraceptive methods as compared to reproductive-age women who did not have any job respectively. The finding is consistent with the finding from secondary data analysis of the 2003–2014 Ghana Demographic and Health Surveys [17]. The results of the descriptive study also depicted that 24.65, 23.89, 29.01 and 24.44% of reproductive age women utilized modern contraceptive method from sales, agricultural employee, skilled manual working and others respectively, whereas, only 16.61% utilized modern contraceptive method from those who did not have any job. This could be due to the reason that women who are employed and have their private job do have better income, better access to media and health facilities which positively influence modern contraceptive method utilization [14, 19, 21, 23, 25, 26]. Besides, they may need to have a gap between bearing children as they are on duty. Some women may be the only breadwinners for their families and might frequently work to sustain the lives of their families.

The number of children was also independently associated with the utilization of modern contraceptive methods. A woman who had no child and who had 1–2 child/children was nearly 75 and 34% times less likely utilized modern contraceptive method as compared to a woman who had born 5 or more children. This finding is in line with the results of secondary data analysis of 2012 and 2015 Afghanistan Demographic and Health Survey where women with more than 6 children more likely utilized modern contraceptive methods [21, 23]. This may be due to the reason that women with a fewer number of children may need to bear more children to attain the desired family size [27]. But the current result is inconsistent with the result of secondary data analysis of three consecutive Bangladesh Demographic and Health Surveys where women with 4 or more children less likely utilized modern contraceptive methods as compared to women who had 0–1 child [22]. The possible reason for this discrepancy could be the socio-economic or socio-cultural difference between the two countries which might have an impact on the number of children desired. The result was also discordant with the result of the cross-sectional study done in Debre Birhan District of north Shoa zone, Ethiopia in 2010 where women who had more than five children less likely utilized modern contraceptive methods as compared to women who had no child [12]. This difference might have occurred due to the difference in the number of study participants participated in the study; the current study used a relatively large sample size which may better reflect the true population parameter as compared to any other study done with a small sample size.

The husband’s educational status was positively associated with modern contraceptive method utilization. A woman whose husband had completed primary education was nearly 1.4 times more likely to utilize the modern contraceptive method as compared to a woman whose husband was not educated. The finding is consistent with the evidence from the 2014 Bangladesh Demographic and Health Survey where a woman whose husband completed higher education 1.28 times more likely utilized modern contraceptive methods as compared to a woman whose husband was not educated [28]. The finding was also concordant with the finding from a community-based cross-sectional study done at rural Dembia District, northwest Ethiopia in 2015 where a woman who had uneducated husband had 72% less likely utilized modern contraceptive method as compared to a woman who had a husband who completed grade 7 and above [10]. This could be due to the reason that educated husbands might have a good insight on modern contraceptives and compromise unreasonable social norms, beliefs, and attitudes towards modern contraceptive utilization and encourage their wives to utilize them. Besides, educated husbands may better share decision-making autonomy with their wives and approve the utilization of modern contraceptive utilization. Husband’s education is also related to better household income which has a positive impact on modern contraceptive utilization [14, 19, 26].

Multivariable logistic regression also showed that age at first sexual intercourse was independently associated with modern contraceptive utilization. A woman who had started her first sexual intercourse at an age of less than 18 years was nearly 1.6 times more likely to utilize the modern contraceptive method as compared to reproductive-age woman who started her first sexual intercourse at an age of 25 or more year respectively. This may be due to the reason that women under 18 years of age are, most of the time, economically dependent and do not want to bear a child and use a contraceptive to delay pregnancy. In the Ethiopian context, usually, reproductive age woman under 18 years of age wants to pursue her education and use a contraceptive to delay pregnancy. But this result is inconsistent with the result of the cross-sectional study done among sexually active Nepal youths, where those who started their first sexual intercourse between 12 and 15 years of age were less likely to use modern contraceptive as compared to those aged 20 to 24 year [29]. The difference could be due to the reason that the study done in Nepal considered only sexually active adult youths where the current study was done on all reproductive-age women aged 15 to 49 years.

The current study also revealed that the husband’s desire for more children was inversely associated with modern contraceptive utilization. The odds of modern contraceptive utilization among women whose husbands want more children was nearly 25% less likely than the odds of modern contraceptive utilization among women who want the same number of children with their husbands. This is consistent with the evidence from Bangladesh, Burkina Faso and Mali secondary data analysis of Demographic and Health Survey where husband’s desired number of children had influenced modern contraceptive utilization among reproductive-age women [20, 28]. This could be due to male dominance in decision making autonomy in developing countries including our country, Ethiopia. When decisions are not equally made on the required family size in the household, males dominate the decision making power and decide on the number of the family size desired. Therefore, if husbands aspire for more children than their wives, they may negatively influence their spouses on utilizing family planning.

The current study also revealed that the wealth index was independently associated with current contraceptive utilization. A woman who was from the richest, a richer, middle and poorer household was nearly 3.5, 3, 2.5 and 2 times more likely to utilize modern contraceptive methods as compared to reproductive age woman from the poorest household respectively. This finding is consistent with findings from secondary data analysis of Demographic and Health Survey in Afghanistan in 2012 and 2015 [21, 23] and secondary data analysis of Demographic Health Survey in Nigeria in 2013 [19] where richest women more likely utilized modern contraceptive method as compared to poor women. The finding is also consistent with the results of a cross-sectional study conducted in Malawi in 2010 [26] and a cross-sectional study conducted in Nigeria in 2016 [25]. A cross-sectional study conducted in western Ethiopia in 2014 also showed that a woman who had a monthly income of 1001–1500 ETB per month had nearly 2 times more likely utilized modern contraceptive methods as compared to a woman who earned less than 600EBR per month [14]. This might be due to the reason that the richest woman has more access to media and health facilities and might have a better awareness of modern contraceptive methods than those from a poor family [14, 19, 26].

In the multivariable analysis, watching television was also independently associated with modern contraceptive utilization among reproductive-age women. A woman who watches television at least once a week and less than once a week was nearly 1.3and 1.4 times more likely to utilize modern contraceptive methods as compared to a woman who does not watch television. The finding is consistent with the evidence from secondary data analysis of 2012 and 2015Afghanistan Demographic and Health Survey, 2014 Bangladesh Demographic and Health Survey and 2013 Nigeria Demographic and Health Survey where women who had media exposure utilized modern contraceptive method more likely as compared to women who had no media exposure [21–23, 25]. This may be due to the reason that women who had media exposure might have a better awareness of modern contraceptives and their utilization. But a cross-sectional multi-country analysis of Demographic and Health Surveys (DHS) conducted between 2008 and 2016 in 52 low and middle-income countries (LMICs) showed that there is no association between exposure to media and modern contraceptive utilization among young reproductive-age women [15]. The difference might have occurred due to the age difference between the study participants; the current study included all reproductive-age women, whereas, the later one only included young reproductive-age women aged between 15 and 24 years. Young reproductive age women might not give due attention to the messages conveyed through media and benefitted from it.

The current study has its strengths and limitation. The study used nationally weighted representative data which better reflects the proportion of reproductive-age women using modern contraceptive method and its associated factors at the national level. Since the study used data from a single time survey, the temporal relationship between modern contraceptive use and the aforementioned predictor variables cannot be assured and the evidence should be utilized with caution. Besides, because of the lack of qualitative data on EDHS data, the association of qualitative variables like socio-cultural factors to modern contraceptive utilization was not addressed in the current study.

In the current study, a woman was considered as modern contraceptive method utilizer if she had been using at least one of the modern contraceptives (female sterilization, male sterilization, IUCD, injectable, implants, pills, male condom, female condom, emergency contraception, and standard days method) only during EDHS data collection period. Therefore, since it was a single time survey, it does not show variations in the utilization of modern contraceptive methods by reproductive-age women in Ethiopia by period and results must be used with caution.

## Conclusions

The magnitude of modern contraceptive utilization among reproductive-age women in Ethiopia in the current study is unexpectedly low. Age, residence, region, woman’s occupation, number of living children, husband’s education, age at first sexual intercourse, husband’s desire for more children, wealth index and watching TV were independent predictors of modern contraceptive use among reproductive-age women in Ethiopia. Any intervention strategy that promotes modern contraceptive method utilization should consider these factors for its better success. Future researchers interested in the area should also address qualitative variables like socio-cultural factors which might have a tremendous effect on modern contraceptive utilization.

## Data Availability

The SPSS datasets used and/or analyzed during the current study are available from the corresponding author on reasonable request.

## Abbreviations

AOR: Adjusted Odds Ratio
CI: Confidence interval
COR: Crude Odds Ratio
CSA: Central Statistical Agency
EDHS: Ethiopia Demographic and Health Survey
ETB: Ethiopian Birr
FMoH: Federal Ministry of Health
LMIC: Low and Middle Income Country
SPSS: Statistical Package for Social Science
